# Quality of life and colorectal function in Crohn’s disease patients that underwent ileocecal resection during childhood

**DOI:** 10.1007/s00431-019-03427-3

**Published:** 2019-07-20

**Authors:** Kay Diederen, Lissy de Ridder, Patrick van Rheenen, Victorien M. Wolters, Maria L. Mearin, Tim G. de Meij, Herbert van Wering, Matthijs W. Oomen, Justin R. de Jong, Cornelius E. Sloots, Marc A. Benninga, Angelika Kindermann

**Affiliations:** 1 Department of Pediatric Gastroenterology and Nutrition, Emma Children’s Hospital, Academic Medical Center, Meibergdreef 9 (room C2-312), 1105 AZ Amsterdam, The Netherlands; 2 Department of Pediatric Gastroenterology, Erasmus MC-Sophia Children’s Hospital, Rotterdam, the Netherlands; 3 Dept. of Pediatric Gastroenterology, University of Groningen, University Medical Centre Groningen, Groningen, the Netherlands; 4 Department of Pediatric Gastroenterology, University Medical Center Utrecht, Utrecht, the Netherlands; 5 Department of Pediatrics, Leiden University Medical Center, Leiden, the Netherlands; 6 Department of Pediatric Gastroenterology, Amsterdam University Medical Centers - VUmc, Amsterdam, the Netherlands; 7 Department of Pediatrics, Amphia Hospital, Breda, the Netherlands; 8 Department of Pediatric Surgery, Amsterdam University Medical Centers - AMC, Emma Children’s Hospital, Amsterdam, the Netherlands; 9 Department of Pediatric Surgery, Erasmus MC-Sophia Children’s Hospital, Rotterdam, the Netherlands

**Keywords:** Pediatric, Crohn’s disease, Surgery, Intestinal resection, Quality of life, Colorectal function.

## Abstract

Psychosocial and functional outcomes after intestinal resection in pediatric Crohn’s disease (CD) are lacking. Therefore, we (I) assessed health-related quality of life (HRQOL), colorectal function, and satisfaction with surgery and (II) investigated their relationship with surgical outcomes, after ileocecal resection for CD. Crohn’s patients that underwent ileocecal resection during childhood were included. HRQOL and colorectal function were assessed using *SF-36* and *COREFO*, respectively, and compared with reference values. Satisfaction was scored on a 5-point Likert scale. In total, 80 patients (50% male, median age 23.0 years) were included. Physical HRQOL was impaired (SF-36 [mean]: CD, 47 vs. general, 54; *p* < 0.001), while mental HRQOL was similar to that in the general population. Overall colorectal function was impaired (COREFO [mean]: CD, 12.6 vs. normal, 7.2; *p* < 0.001). Worse colorectal function was associated with increasing clinical disease activity and longer interval since resection. Majority of patients was satisfied with surgery (81% satisfied/very satisfied, 11% neither satisfied nor dissatisfied, 8% dissatisfied/very dissatisfied). Decreased satisfaction with surgery was associated with increased clinical disease activity but not related to colorectal function.

*Conclusions*: Physical HRQOL and colorectal function in CD patients who underwent ileocecal resection during childhood seem impaired and related to adverse surgical outcomes. This emphasizes the need for post-operative monitoring and prophylactic therapies.

**Table:** 

**What is Known:** *• Up to 25% of pediatric-onset Crohn’s disease (CD) patients undergo an intestinal resection within 5 years from diagnosis.* *• Many children and adults with CD experience disruption of their daily activities and health-related quality of life (HRQOL).*	
**What is New:** *• Physical HRQOL and colorectal function are impaired in patient with CD that underwent ileocecal resection during childhood.* *• Increasing clinical disease activity, a longer interval since surgery, severe complications related to surgery, and recurrent surgeries are all associated with worse colorectal function.*

**What is Known:**

*• Up to 25% of pediatric-onset Crohn’s disease (CD) patients undergo an intestinal resection within 5 years from diagnosis.*

*• Many children and adults with CD experience disruption of their daily activities and health-related quality of life (HRQOL).*

**What is New:**

*• Physical HRQOL and colorectal function are impaired in patient with CD that underwent ileocecal resection during childhood.*

*• Increasing clinical disease activity, a longer interval since surgery, severe complications related to surgery, and recurrent surgeries are all associated with worse colorectal function.*

## Introduction

Crohn’s disease (CD) is a chronic, recurrent bowel disease, characterized by patchy, transmural inflammation which can involve any segment of the gastrointestinal tract [21]. In 7% to 20% of cases, CD is already diagnosed during childhood [5]. Up to 25% of pediatric-onset CD patients undergo resection within 5 years from diagnosis [16]. The majority of these procedures concern ileocecal resection, especially for disease confined to the ileocecal region [20].

Irrespective of the surgical treatments, many children and adolescents with CD experience disruption of their daily activities and health-related quality of life (HRQOL) [6, 11, 33]. Inducing remission by ileocecal resection is associated with significant short-term improvements of HRQOL in adults with CD [12, 28]. However, the endurance of improved HRQOL following surgery in the long run is contentious [12, 28]. Post-operative complications and clinical recurrence are shown to adversely affect HRQOL in adults with CD post-operatively [28, 31]. Data on children with CD are limited to a small cohort with intestinal resections of different extent and location [27]. Besides, it is uncertain whether ileocecal resection impacts colorectal function. In patients with extensive ileal and colonic resections, a loss of absorptive capacity for fat, bile acids, and sodium chloride can lead to diarrhea [2, 14, 19]. Another study including adults with varying resections of ileum and/or colon observed that resection of the ileocecal valve and parts of the ascending colon was associated with more diarrhea compared with resection of the terminal ileum alone [25]. Although children with CD tend to undergo limited resections, indeed the ileocecal valve is resected too. It is unknown if these patients encounter chronic diarrhea likewise.

Therefore, this study aimed to (I) assess HRQOL and colorectal function, (II) investigate the relation between colorectal function and surgical characteristics or adverse outcomes, and (III) appraise satisfaction with the surgical procedure after primary ileocecal resection in pediatric CD patients.

## Materials and Methods

### Patients

In this cross-sectional study, we included all patients with an established diagnosis of CD according to the (revised) Porto criteria [23], who underwent primary ileocecal resection for CD during childhood (age < 18 years) between January 1990 and December 2014 in one of six tertiary hospitals in the Netherlands (Academic Medical Center, Erasmus Medical Center, University Medical Center Groningen, University Medical Center Utrecht, Leiden University Medical Centre, VU University Medical Center). Primary ileocecal resection was defined as laparoscopic or open ileocecal resection as first surgery for CD without a history of other abdominal resections, except appendectomy. All procedures were performed by experienced pediatric surgeons. Complications, disease recurrence, and anthropometrics in this cohort have been published previously [9]. The study was approved by the medical ethics committees and informed consent was given.

### Data collection

Patients were identified from institutional databases covering all types of surgical procedures. Patients were contacted by mail and asked to complete questionnaires on clinical disease activity, HRQOL, colorectal function, and satisfaction with surgery. If questionnaires were not returned within 6 weeks, patients were contacted by telephone (maximum of three attempts), by an investigator (K.D.) independent of their healthcare team. Eligible patients who declined participation or did not respond to mail and telephone were defined as non-responders and excluded.

#### Patient characteristics, surgical details, and disease course

Patient characteristics, surgical details, and disease course after ileocecal resection were obtained from medical records, including age, sex, disease phenotype (Paris classification) [22], CD-related medication, type of approach and anastomosis, primary stoma rate, operating time, and pathology details.

Disease course after ileocecal resection included severe complications and recurrent abdominal surgery. Severe post-operative complications were defined as a Clavien-Dindo classification grade ≥ III (requiring surgical, endoscopic or radiological intervention) [9, 10]. Recurrent abdominal surgery was defined as disease recurrence requiring new resection for active inflammation or strictureplasty for (anastomotic) strictures [9].

#### Clinical disease activity

Clinical disease activity in patients who reached adulthood (≥ 18 years) was determined using the Harvey-Bradshaw Index (HBI) [17]. The HBI consists of 5 domains, with cut-off scores for remission (≤ 4), mild (5–7), moderate (8–16), and severe disease (≥ 17) [17]. Clinical disease activity in pediatric patients (< 18 years) was determined using the abbreviated Pediatric Crohn’s Disease Activity Index (aPCDAI) [24, 29]. The aPCDAI consists of six clinical items, with cut-off scores for remission (≤ 10 points), mild (11–25), moderate (26–39), and severe disease (≥ 40) [24, 29]. Moderate and severe diseases were analyzed as one group, due to the expected small group size.

#### Health-related quality of life

Quality of life was evaluated only in patients who reached adulthood, as there are no validated tools available that can reliability assess HRQOL in both adults and children. Adults completed the Dutch version of the short form health survey 36 (SF-36) [1], a valid instrument for HRQOL assessment in both healthy and chronically ill adults. Scores were aggregated into two summary measures: Physical and Mental Component Summary [34]. Scale scores range from 0 to 100, with higher scores representing better HRQOL. Mean SF-36 scores from adult patients were compared with pre-published mean scores of adults between the age of 26 and 35 years from the Dutch general population, which were used to validate and norm the Dutch language version of the SF-36 [1].

#### Colorectal function

The colorectal function outcome (COREFO) questionnaire is a validated questionnaire with 27 questions to assess colorectal function [3]. The COREFO questionnaire assesses five categories: incontinence, social impact, defecation frequency, stool-related aspects (pain during bowel movements, blood loss, and local skin problems), and use of medication to thicken bowel movements. Scores per category and total score range from 0 to 100, with higher scores representing worse outcomes. Mean COREFO scores were compared with the pre-published mean score of individuals with normal colorectal function, consisting of individuals who had a laparoscopic cholecystectomy, used in the validation study of the COREFO questionnaire [3].

#### Satisfaction with surgery

Satisfaction after primary ileocecal resection was measured asking the question *“*how satisfied are you with the performed surgery (primary ileocecal resection)?.” Answers were given on a 5-point Likert scale: very satisfied, satisfied, not satisfied nor dissatisfied, dissatisfied, and very dissatisfied.

### Statistical analysis

Patient, disease, and surgical variables between responders and non-responders were compared. Continuous data were presented as mean and standard deviation or median and interquartile range (IQR) and unpaired *T* tests and ANOVA with Tukey’s multiple comparison post hoc test.

The Crohnbach α’s of the SF-36 and COREFO scores were calculated [13]. Crohnbach’s α ≥ 0.60 was considered acceptable, due to the diversity of items per scale and the small number of items in some subscales [13]. Cronbach’s α for all SF-36 items was 0.68. Cronbach’s α for the COREFO total score was 0.88, with subscores ranking between 0.52 and 0.82, except for the medication subscale (0.39). The COREFO categories with α’s under the set threshold (i.e., medication, stool frequency, incontinence) can be explained by the few items (two to three) in all of these categories.

HRQOL scores and COREFO total and category scores of the reference cohort were compared to all CD patients, and those with disease in clinical remission (HBI ≤ 4 [17], aPCDAI ≤ 10 [24, 29]) and active disease (HBI > 4 [17], aPCDAI > 10 [24, 29]) using unpaired *T* tests or ANOVA with Tukey’s multiple comparison post hoc test, on mean, standard deviation (SD), and number of subjects.

Associations between colorectal function (COREFO total and category scores) and clinical disease activity (categorized: remission, mild, moderate-severe), time since ileocecal resection, length of resected ileum (excluding the cecum), and occurrence of severe complications and recurrent abdominal surgery were identified by univariate linear regression. Associations between satisfaction with surgery (5-point Likert scale) and clinical disease activity (categorized: remission, mild, moderate-severe), time since ileocecal resection, or age at follow-up were also identified by univariate linear regression. Non-normally distributed continuous variables were first normalized by natural logarithm. Variables with a two-sided *p* value < 0.10 in univariable regression were included in multivariate analysis, additionally corrected for sex and age at follow-up.

Effect sizes were computed according to Cohen’s *d* and categorized as small (0.2–0.5), medium (0.5–0.8), and large (> 0.8) [4]. Statistical analysis was performed using IBM SPSS Statistics 22 and GraphPad Prism 5 for Windows. All statistical tests were two-sided and assessed at a significance level of 1% for the difference in HRQOL and colorectal function (to adjust for multiple testing) and at 5% for all other tests.

## Results

A total of 114 patients underwent primary ileocecal resection, of whom 80 patients (50% male, median age at follow-up 23.0 years) were included (response rate 70.1%). Responders and non-responders did not differ on sociodemographic variables, disease characteristics, surgical details, and time of follow-up (variables of responders are exhibited in Table 1). Indications for ileocecal resection were stenosis of the ileocecal area (64%), and/or therapy refractory inflammation (28%), and/or intra-abdominal fistulae or abscesses (26%).

**Table 1:** Demographic and surgical characteristics of pediatric Crohn’s disease patients at the time of ileocecal resection

	Responders (*n* = 80)
Male (*n*, %)	50 (50%)
Age, surgery (median, IQR)	15.0 (14.0–16.0)
Age, follow-up (median, IQR)	23.0 (18.5–30.0)
Age at diagnosis^a^	
A1a, < 10 years (*n*, %)	7 (9%)
A1b, 10–17 years (*n*, %)	73 (91%)
Disease location, terminal ileum—colon^a^	
L1, distal 1/3 ileum ± limited cecum (*n*, %)	49 (61%)
L2, colonic (*n*, %)	0 (0%)
L3, ileocolonic (*n*, %)	31 (39%)
Disease location, upper gastrointestinal^a,b^ (L4a/L4b)	25 (31%)
Disease behavior^a^	
B1, non-stricturing, non-penetrating (*n*, %)	14 (18%)
B2, stricturing (*n*, %)	32 (40%)
B3, penetrating (*n*, %)	9 (11%)
B2B3, stricturing and penetrating (*n*, %)	25 (31%)
Perianal disease^a^ (*n*, %)	18 (23%)
Medical therapy ever used before surgery	
Steroids (*n*, %)	62 (78%)
Anti-TNF-α (*n*, %)	30 (38%)
Immunomodulators (*n*, %)	59 (74%)
Mesalazine (*n*, %)	35 (44%)
Surgical access	
Open (*n*, %)	32 (42%)
Laparoscopy (*n*, %)	44 (58%)
Anastomosis, type	
End-to-end (*n*, %)	55 (68%)
End-to-side (*n*, %)	5 (7%)
Side-to-side (*n*, %)	16 (20%)
Anastomosis, suture technique	
Hand-sewn (*n*, %)	64 (88%)
Stapled (*n*, %)	9 (12%)
Primary ileostomy (*n*, %)	2 (2.5%)
Additional procedures (*n*, %)	9 (11%)
Resection specimen	
Length (cm) (median, IQR)	24.0 (18.0–32.0)
Resection margin positivity^c^ (*n*, %)	44 (55%)
Emergency surgery (*n*, %)	3 (4%)
Months of follow-up (median, IQR)	81.0 (20.5–163.0)

^a^According to the Paris classification

^b^L4a, upper disease proximal to ligament of Treitz; L4b, upper disease distal to ligament of Treitz and proximal to distal 1/3 ileum

^c^Microscopically positive resection margins

*anti-TNFα*, anti-tumor necrosis factor alpha

Variables containing missing data in included patients (*n* = 80): preoperative medication *n* = 1 (1%); immunomodulators *n* = 1 (1%); mesalazine *n* = 1 (1%); access *n* = 4 (5%); anastomosis, type *n* = 4 (5%); anastomosis, suture technique *n* = 7 (9%); resection specimen (length) *n* = 1 (1%); resection specimen (resection margin positivity) *n* = 7 (9%), emergency surgery *n* = 1 (1%)

Based on clinical indices, 58 (73%) patients were in remission, 12 (15%) had mild disease activity, and 10 (13%) had moderate to severe disease activity at follow-up. At the time of follow-up, 66 patients reached adulthood, and 14 were still below the age of 18 years. None of the patients died during follow-up. The median time of follow-up after primary ileocecal resection was 81.0 months (IQR 20.5–163.0).

### Health-related quality of life

**Fig. 1:**
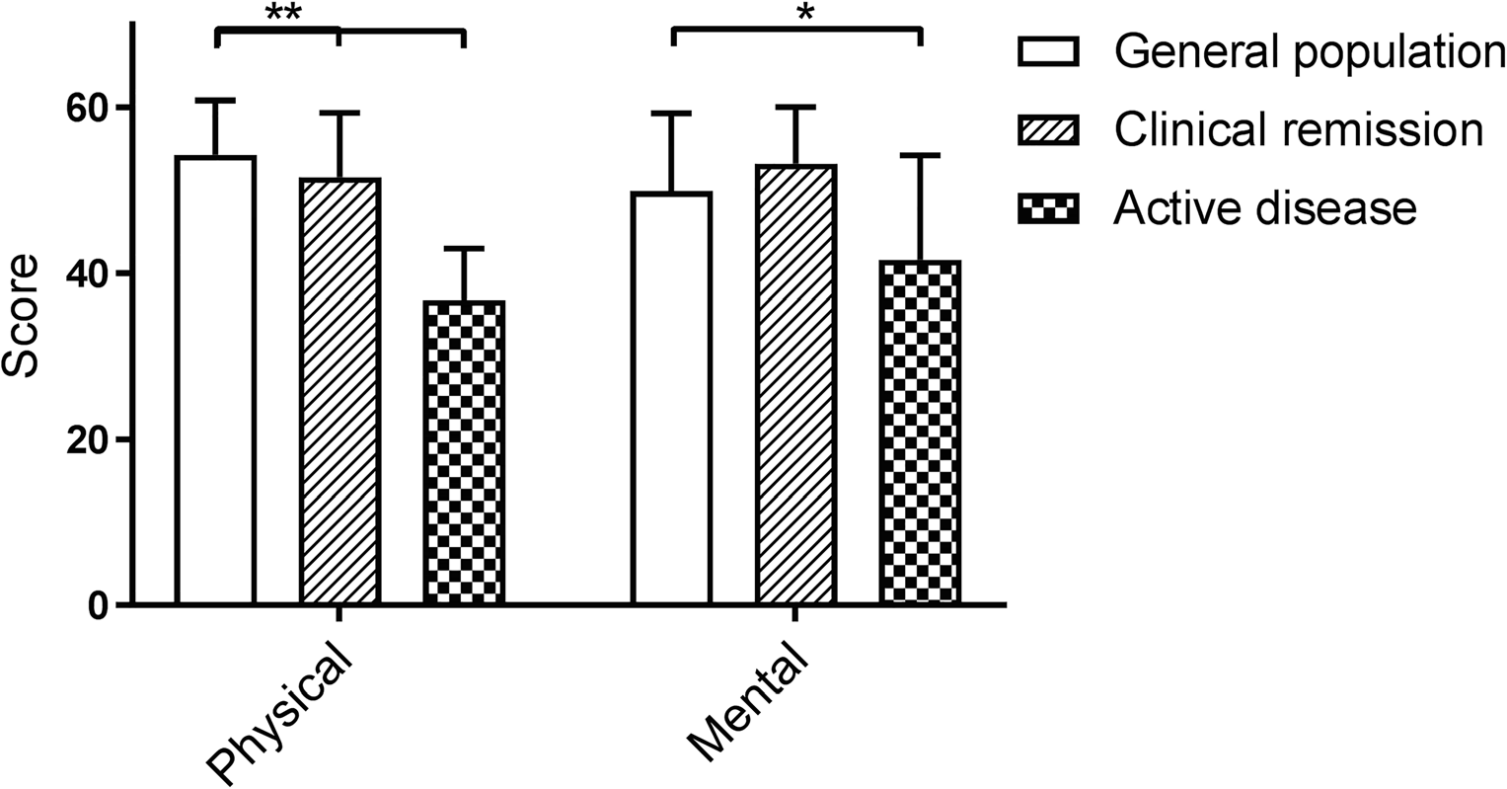
Health-related quality of life measured with the short form 36 in patients with ileocecal resection in clinical remission and with active disease, compared with subjects in the general population

As mentioned in the “Materials and methods” section, quality of life was assessed in patients that reached adulthood at time of study (66/80 patients (83%), 56% male, median age at follow-up 23.5 years (IQR 20.2–31.9), and median follow-up 102.9 months (IQR 46.5–190.0). Adults with CD who underwent primary ileocecal resection have a lower physical health score compared with the general adult population (mean [SD], 46.9 [10.0] vs. 54.3 [6.5], Cohen’s *d* 0.88, *p* < 0.001). Mental health summary scores did not significantly differ (mean [SD], CD 48.5 [10.5] vs. general 49.9 [9.4], *p* = 0.779, Cohen’s *d* 0.04). When CD patients were sub-classified based on disease activity, both patients with active disease (*n* = 21, *p* < 0.001) and those in clinical remission (*n* = 45, *p* = 0.027) had a lower physical health score compared with the general population (mean [SD], active 36.8 [6.2] vs. remission 51.5 [7.8] vs. general, 54.3 [6.5]; Fig. 1). Mental health scores did only differ between patients with active disease (*p* < 0.001) and the general adult population (mean [SD], active 41.6 [12.6] vs. remission 53.2 [6.8] vs. general, 49.9 [9.4]; Fig. 1).

### Colorectal function and associated surgical characteristics and outcomes

Ileocecal-resected CD patients had significantly higher COREFO total scores, compared with a normal colorectal function cohort (total COREFO [mean]: CD, 12.6 vs. normal, 7.2; *p* < 0.001). When patients were stratified to clinical remission (*n* = 58) or active disease (*n* = 22), subjects in remission did not significantly differ from a normal colorectal function cohort on the COREFO total score, but only on the social impact category, with medium effect size (Table 2). Subjects with active disease did significantly differ from a normal colorectal function cohort with higher scores for the COREFO total and all category scores, with medium to large effect sizes (Table 2).

**Table 2:** COREFO total and category scores in patients with CD in remission and active disease compared with a cohort with normal colorectal function

	Normal colorectal function mean (SD)	Ileocecal resection			
		Remission mean diff. (95%CI)	Cohen’s *d*	Active disease mean diff. (95%CI)	Cohen’s *d*
Incontinence	5.6 (7.5)	− 2.9 (− 0.1–5.8)	0.46	+ 4.9 (0.7–9.0)*	0.52
Frequency	6.2 (8.8)	+ 2.8 (− 1.4–7.1)	0.33	+17.7 (11.8–23.6)*	1.24
Stool-related aspects	7.7 (12.9)	+ 2.4 (− 4.4–9.1)	0.16	+ 22.2 (12.8–31.7)*	1.05
Need for medication	6.1 (15.6)	+ 1.7 (− 5.7–9.0)	0.11	+ 25.7 (15.4–36.1)*	1.07
Social impact	4.9 (7.9)	+5.8 (1.12–10.5)*	0.60	+ 35.6 (29.1–42.2)*	2.27
Total score	7.2 (7.0)	+ 0.5 (− 2.8–3.8)	0.08	+ 19.4 (14.8–24.0)*	1.79

*COREFO*, colorectal function outcome; *SD*, standard deviation; *95%CI*, 95% confidence interval

*Significant difference (*p* < 0.01)

Higher clinical disease activity was associated with higher COREFO total score (B, 0.27 [95%CI, 0.16–0.39], *p* < 0.001) and all category scores (Table 3). A longer duration since primary ileocecal resection was associated with a higher COREFO total score (B, 0.40 [95%CI, 0.05–0.75], *p* = 0.025), and a higher “defecation frequency” (Table 3). A severe complication after ileocecal resection was associated with a higher “defecation frequency,” and recurrent abdominal surgery with a “higher need of medication” (Table 3). The length of the resected ileum was not associated with COREFO total and category scores (Table 3).

**Table 3:** Association between patient and surgical characteristics and COREFO total and subscores

	Incontinence B (95% CI)	Need for medication B (95%CI)	Frequency B (95%CI)	Stool-related aspects B (95%CI)	Social impact B (95%CI)	Total score B (95%CI)
Clinical disease activity^a^	0.17 (0.02–0.34)*	0.22 (0.01–0.41)*	0.31 (0.14–0.47)*	0.24 (0.02–0.47)*	0.35 (0.19–0.52)*	0.27(0.16–0.39)*
Time since resection (months)	0.46 (− 0.03–0.95)	0.46 (− 0.15–1.08)	0.67 (0.13–1.21)*	0.17 (−0.53–0.88)	0.46 (−0.06–0.97)	0.40 (0.05–0.75)*
Length of resection (cm)	–	–	–	–	–	–
Complications surgery^b^ Yes vs. no	–	0.35 (− 0.09–0.80)	0.40 (0.02–0.79)*	–	–	–
Surgical recurrence^c^ Yes vs. no	0.13 (− 0.22–0.47)	0.52 (0.09–0.95)*	–	0.16 (−0.34–0.65)	0.16 (−0.20–0.53)	0.13 (−0.12–0.37)

*COREFO*, colorectal function outcome

*Significantly associated with multivariable regression analysis, corrected for sex and age at follow-up (*p* < 0.05). Variables with a *p* value of ≥ 0.1 in univariable analysis are indicated by (−) and excluded from multivariable analysis

^a^Categorical disease activity: remission, mild, moderate, and severe.

^b^Clavien-Dindo classification grade ≥ III (requiring surgical, endoscopic, or radiological intervention)

^c^Disease recurrence requiring new resection for active inflammation or strictureplasty for (anastomotic) strictures

### Satisfaction with surgery

Satisfaction with primary ileocecal resection was scored as follows (high–low): 42 (53%) very satisfied, 23 (29%) satisfied, 9 (11%) neither satisfied nor dissatisfied, 4 (5%) dissatisfied, and 2 (3%) very dissatisfied. Higher clinical disease activity was associated with lower satisfaction with surgery (B, − 0.48 [95%CI, − 0.83 to − 0.12], *p* = 0.009). The duration since primary ileocecal resection (B, − 0.27 [95%CI, − 0.98–0.43], *p* = 0.439) was not associated with satisfaction with surgery. Satisfaction with surgery was not related to colorectal function (COREFO total score: B, − 0.067 [95%CI, − 0.15–0.01], *p* = 0.105).

## Discussion

In this multicenter study, we determined HRQOL and colorectal function, investigated the relation between colorectal function and surgical characteristics and outcomes, and appraised satisfaction with the surgical procedure, in patients with CD who underwent primary ileocecal resection during childhood.

Physical HRQOL in adults that underwent ileocecal resection during childhood was decreased compared with that in the general population. To date, one study investigated HRQOL after intestinal resection in children, which reported decreased HRQOL on physical and social domains in patients who had missed school or work, but not in those who could normally attend school or work [27]. Unlike our cohort, this study included patients with a wide variety of intestinal resections (ranging from small bowel resections to full proctocolectomy) and used a visual analog scale to measure HRQOL, the former introducing heterogeneity and all together inhibits adequate comparison. In a study on HRQOL in adults with CD that underwent ileocecal resection, which made use of the same HRQOL tool (i.e., SF-36), physical but not mental HRQOL was impaired, as seen in our cohort [12]. Lower physical HRQOL also corresponds to previous studies in general pediatric inflammatory bowel disease (IBD) populations, which exhibited a decreased physical activity [35].

In our cohort of ileocecal-resected patients, colorectal function was impaired compared with that in a cohort with normal colorectal function. To evaluate if patients with clinically active disease would exhibit impaired colorectal function (i.e., COREFO scores), we stratified patients into those with clinically active or inactive CD. Indeed, patients with CD in clinical remission had an overall colorectal function similar to a cohort with normal colorectal function, barring impairment of social activities due to bowel problems (troublesome urgency and feeling of incomplete evacuation). Substantial problems regarding the feeling of incomplete evacuation were also seen in a small cohort (*n* = 5) of pediatric CD patients who underwent an ileal resection [27]. If this can be related to surgery remains elusive, as feelings of incomplete evacuation are frequently reported in CD patients not subjected to elective surgery [8].

Aspects of colorectal function were associated with clinical disease activity, time since selective surgery, severe complications related to surgery, and recurrent abdominal surgery after primary ileocecal resection.

Higher clinical disease activity was associated with decreased scores on all enquired colorectal function categories. It seems plausible and matches the current literature that patients with active disease after ileocecal resection experience more problems regarding defecation frequency, stool-related aspects, and social impact of colorectal function [12, 28, 36]. In particular, increased incontinence problems are troublesome. Whether incontinence can be related to ileocecal resection is unknown. Bile acid malabsorption, attributed to the reduction in absorptive capacity in patients with extensive ileal resections, has been reported, which may result in diarrhea and fecal continence [2, 19]. In our cohort, which primarily included patients with limited ileum resections, no associations between the length of the resected ileum and incontinence were found.

Interestingly, we observed worse colorectal function with a longer duration since resection, including increased stool frequency. In a previous cohort of adults who underwent elective surgery for CD, stool frequency and other symptoms influencing colorectal function also increased during follow-up [32]. This is likely related to high rates of disease recurrence after elective surgery, which is a major point of concern. Severe complications and recurrent abdominal surgery were associated with higher stool frequency and higher need of medication or food adjustments (for thickening of bowel movements), respectively. In a previous study on adult patients who underwent ileocolonic resections, those with the need of additional abdominal surgery experienced impaired intestinal and systemic functional scores and lower HRQOL [28].

The majority of patients were satisfied or very satisfied with their ileocecal resection (81%). In agreement with our cohort, a previous study reports that 80% of patients were satisfied with surgery and would choose to undergo the procedure again if necessary [7, 30].

This study has several implications. First, physical HRQOL after ileocecal resection for pediatric CD was impaired, indicating the importance of monitoring HRQOL after surgery. Monitoring of HRQOL can consist of periodic screening with a suitable questionnaire, as already performed in our center [18]. Those with psychosocial problems should be referred to psychological health care on individual or group level [26]. Moreover, the impaired HRQOL after surgery should prompt for expectation management in children undergoing this procedure, especially because an ileocecal resection usually does not offer a cure for CD. Secondly, despite quiescent CD, patients still indicated impairment of social activities due to colorectal function. This may suggest that despite a lack of symptoms according to widely used validated clinical indices, patients with CD still experience debilitating symptoms after ileocecal resection. This stresses the use of validated patient-reported outcomes, to reliably measure how children with CD feel and function after elective surgery [15]. Thirdly, this study indicates that adverse surgical outcomes, such as post-operative complications and recurrent surgery, are associated with impaired colorectal function, which emphasizes the need for careful post-operative monitoring and prophylactic therapies. Immediate post-operative therapy after ileocecal resection reduced the risk of both a symptomatic relapse and a second resection [9], with anti-TNF-α likely being most effective [37].

This is the first study that investigated psychosocial and functional outcomes in patients that underwent a primary ileocecal resection during childhood, thereby excluding heterogeneity due to elective surgeries from various extent and allows to stratify for the effect of additional resections. The limitation of this study is the lack of pre- and post-intervention assessment of HRQOL and colorectal function. Therefore, we were not able to assess the exact effect of ileocecal resection on HRQOL, as the cohort was only composed after surgery. This might raise the question whether ileocecal resection itself or other disease characteristics, such as disease behavior and post-operative prophylaxis, are contributing to the decreased HRQOL and worse colorectal function. Results of the effect of ileocecal resection on psychosocial and functional outcomes should be interpreted with caution. Solid conclusions can therefore not be made. In line with our hypothesis, however, a study on adults with CD that evaluated pre- and post-surgery HRQOL showed that ileocecal resection was associated with significant improvement in the quality of life [36]. Another smaller limitation is the variation in time of follow-up after surgery, which may lead to potential bias, since risk of disease recurrence increases with the lapse of time. Therefore, we corrected for time of follow-up in the multivariable model on the relation between adverse surgical outcomes and (psychosocial and functional) outcomes after ileocecal resection. Moreover, the HRQOL (SD-36) and colorectal function questionnaire (COREFO) cannot perfectly distinguish symptoms related to the resection form symptomen due to active IBD or consequences directly related to the resection. Therefore, we investigated HRQOL and colorectal function in both patients in clinical remission and in those with active disease.

Physical HRQOL and colorectal function are impaired in CD patients who underwent ileocecal resection during childhood. Despite quiescent CD, patients experience debilitating social impact due to impaired colorectal function. Higher clinical disease activity and adverse surgical outcomes are associated with impair colorectal function. This emphasizes the need for careful post-operative monitoring and prophylactic therapies in children with CD.

## Abbreviations

anti-TNFα: Anti-tumor necrosis factor alpha
aPCDAI: Abbreviated Pediatric Crohn’s Disease Activity Index
BMI: Body mass index
CD: Crohn’s disease
COREFO: Colorectal function outcome
CRP: C-reactive protein
HBI: Harvey-Bradshaw Index
HRQOL: Health-related quality of life
IASC: Intra-abdominal septic complications
IBD: Inflammatory bowel disease
PedsQL: Pediatric Quality of Life Inventory 4.0
SF-36: Short form health survey 36
